# Neonatal and infant outcomes of clozapine exposure in pregnancy: A consecutive case series

**DOI:** 10.1192/j.eurpsy.2021.1280

**Published:** 2021-08-13

**Authors:** M.L. Imaz, S. Lera, B. Sureda, A. Roca, S. Andres, A. Giménez Palomo, E. Solé, A. Torres, L. Garcia-Esteve

**Affiliations:** 1 Unit Of Perinatal Mental Health, Department Of Psychiatry And Psychology, Idibaps, University Of Barcelona (ub), Hospital Clinic Barcelona, Barcelona, Spain; 2 Child And Adolescent Psychiatry And Psychology & Unit Of Perinatal Mental Health, Hospital Clinic Barcelona, Barcelona, Spain; 3 Unit Of Perinatal Mental Health, Department Of Psychiatry And Psychology, Hospital Clinic Barcelona, Barcelona, Spain; 4 Unit Of Perinatal Mental Health, Department Of Psychiatry And Psychology, Idibaps, Hospital Clinic Barcelona, Barcelona, Spain

**Keywords:** clozapine, neurodevelopment, Neonate, Infant

## Abstract

**Introduction:**

Clozapine is a second-generation antipsychotic agent approved for treatment-resistant schizophrenia and risk reduction of recurrent suicidal behavior in schizophrenia and schizoaffective disorder. Given the known negative consequences of relapse of severe mental disorders for both mother and infant, the maintenance of clozapine during pregnancy is recommended.^1^ Studies of pregnancy regarding to clozapine have demonstrated a heterogenous range of neonatal and infant complications.^2^

**Objectives:**

To evaluate neonatal and infants outcomes of clozapine exposure in pregnancy.

**Methods:**

We report three cases of infants exposed to clozapine politherapy throughout pregnancy. The dose range for all women on clozapine was 200-600 mg/day. Infants were evaluated between 4-6 months of chronological age with the Bayley-III infant development scale (BSID-III)^3^ and with the Alarme Détresse Bébé Scale (ADBB)^4^ for the detection of early-signs of withdrawal.

**Results:**

Women remained stable during pregnancy but presented obesity and gestational diabetes. Clozapine Newborn were born to term by caesarean section due to breech presentation (N=2) or instrumental delivery due to loss of fetal well-being (N=1). They presented normal weight (3500-3800 gr). Two presented Apgar_min1-5_ 9/10 and one Apgar_min1-5_ 6/8 which showed lethargy and low alertness during the first weeks of life. All showed normal capacity for sociability, reciprocity and development of language and communication. However, one baby had scores in the low normal zone for cognition and another for motor skills.

**Conclusions:**

The infant’s risks of clozapine exposure during pregnancy should be discussed with women and weighed against those associated with other treatments and/or with untreated severe mental illness.

